# Clinically immune hosts as a refuge for drug-sensitive malaria parasites

**DOI:** 10.1186/1475-2875-7-67

**Published:** 2008-04-25

**Authors:** Eili Y Klein, David L Smith, Maciej F Boni, Ramanan Laxminarayan

**Affiliations:** 1 Resources for the Future, 1616 P St., NW, Washington, DC 20036, USA; 2 Department of Ecology and Evolutionary Biology, Princeton University, Princeton, NJ 08544, USA; 3 Department of Zoology, University of Florida, Gainesville, FL 32611, USA; 4 Princeton Environmental Institute, Princeton University, Princeton, NJ 08544, USA

## Abstract

**Background:**

Mutations in *Plasmodium falciparum *that confer resistance to first-line antimalarial drugs have spread throughout the world from a few independent foci, all located in areas that were likely characterized by low or unstable malaria transmission. One of the striking differences between areas of low or unstable malaria transmission and hyperendemic areas is the difference in the size of the population of immune individuals. However, epidemiological models of malaria transmission have generally ignored the role of immune individuals in transmission, assuming that they do not affect the fitness of the parasite. This model reconsiders the role of immunity in the dynamics of malaria transmission and its impact on the evolution of antimalarial drug resistance under the assumption that immune individuals are infectious.

**Methods:**

The model is constructed as a two-stage susceptible-infected-susceptible (SIS) model of malaria transmission that assumes that individuals build up clinical immunity over a period of years. This immunity reduces the frequency and severity of clinical symptoms, and thus their use of drugs. It also reduces an individual's level of infectiousness, but does not impact the likelihood of becoming infected.

**Results:**

Simulations found that with the introduction of resistance into a population, clinical immunity can significantly alter the fitness of the resistant parasite, and thereby impact the ability of the resistant parasite to spread from an initial host by reducing the effective reproductive number of the resistant parasite as transmission intensity increases. At high transmission levels, despite a higher basic reproductive number, *R*_0_, the effective reproductive number of the resistant parasite may fall below the reproductive number of the sensitive parasite.

**Conclusion:**

These results suggest that high-levels of clinical immunity create a natural ecological refuge for drug-sensitive parasites. This provides an epidemiological rationale for historical patterns of resistance emergence and suggests that future outbreaks of resistance are more likely to occur in low- or unstable-transmission settings. This finding has implications for the design of drug policies and the formulation of malaria control strategies, especially those that lower malaria transmission intensity.

## Background

Malaria is the leading cause of death in children under five in sub-Saharan Africa [1]. Prompt treatment with effective antimalarial drugs could prevent much of the morbidity and mortality associated with clinical malaria, but the evolution of resistance has diminished the therapeutic efficacy of two previous first-line antimalarials, chloroquine (CQ) and sulphadoxine-pyrimethamine (SP). Historically, it has been suggested that resistance to both CQ and SP emerged from a limited number of *de novo *selection events in areas of low or unstable transmission [2,3]. Genetic evidence suggests that most CQ-resistant parasites in the world today are descended from one of four founder events [4,5] that occurred in Southeast Asia, South America and Papua New Guinea, and then spread to other regions, including sub-Saharan Africa. Evidence also suggests that resistance to SP originated from only a few foci [6-8]. Several hypotheses about the *de novo *mutation rate and the selection pressure resistant parasites face and their relation to transmission intensity have been proposed to explain why CQ resistance originated in what is presumed to be low or unstable transmission areas outside of sub-Saharan Africa, including (a) a lower frequency of resistant alleles in higher transmission areas because of within-host competition [9]; (b) less drug treatment (per parasite) in higher transmission areas [2,9,10]; and (c) a lower frequency of selfing in higher transmission areas, which increases the probability that multilocus resistant genotypes will be broken up by the action of Mendelian segregation [9,11,12]. An additional explanation is that in high transmission areas, where immunity is better developed, mutant parasites are less likely to survive a host immune response [10,13].

Although the evolution of resistant parasites within a single host is a significant risk factor for the emergence of resistance within a population, the effectiveness of an antimalarial drug is affected only if these resistant parasites spread, a process that is driven both by the overall rate of transmission and by the relative fitness of drug-resistant and drug-sensitive parasites. Parasite fitness is related to the population treatment rate and to the biological cost of resistance. Because of differences in vector ecology and biting preferences, transmission intensities vary from less than one infectious bite per decade to more than 1,000 per year [14], and the basic reproductive number (*R*_0_) can exceed 3,000 [15]. Higher transmission intensity is associated with a higher level of clinical immunity to malaria – reduced frequency and severity of clinical symptoms in older children and adults – which results in a reduction in the need for antimalarial drugs [16-19]. Immunity to malaria has consequences for transmission as well; blood-stage immunity reduces asexual parasite and gametocyte densities in older children and adults [20], and transmission-blocking immunity can block development of the parasite in the mosquito [21]. Importantly, despite significant reductions in clinical symptoms and infectiousness, older individuals still become infected and remain infectious to mosquitoes, albeit at relatively lower levels, even in holoendemic areas [22-24].

Even though immunity affects both the transmission dynamics and treatment rates, epidemiological models have generally assumed that immune individuals are not infectious to mosquitoes, and thus do not contribute to parasite fitness [20,25-27]. Consequently, an epidemiological model for the spread of resistance found no difference in the ability of resistant parasites to spread at different transmission rates [27], or why resistance to CQ emerged outside of sub-Saharan Africa.

This paper reconsiders the role of immunity in the transmission dynamics of malaria and its effect on the evolution of antimalarial drug resistance under the assumption that immune individuals are infectious. Using a novel epidemiological model with two immune stages, nonimmune and clinically immune, the role of immunity in the emergence of resistance is reexamined based on quantitative effects associated with clinical immunity. Individuals in the clinically immune stage have lower transmission, lower incidence of clinical disease, and consequently a lower rate of drug treatment. Based on the defined relationship between transmission intensity, immunity, and clinical malaria, the model is used to explore the relationship between vector ecology, human epidemiology, and the ability of a resistant parasite to spread.

## Methods

Individuals living in areas of endemic *P. falciparum *transmission develop immunity to malaria with age [28] and exposure [29,30]; immunity is manifest as a decline in parasite densities (both trophozoites and gametocytes) in the blood [20], a lower probability of transmission from humans to mosquitoes [22,23], and a decline in the frequency and severity of clinical malaria [16-19], though little decline in the probability of becoming infected [24,31]. Thus, it is assumed that individuals develop a form of clinical immunity over time in which they are less likely to infect mosquitoes or manifest clinical symptoms but are no less likely to become infected.

The model is based on earlier models developed for the Garki Project [20], in which individuals acquire immunity after being infected for a period of time. However, the number of infected classes was simplified and the assumption that individuals develop full transmission-blocking immunity was relaxed. As with the Garki model, clinical immunity is incorporated as a second immune stage in a susceptible-infected-susceptible (SIS) model. Though, in this model clinically immune individuals remain infectious to mosquitoes and infect mosquitoes with lower probability; also, a smaller fraction of new infections progress to clinical malaria. Clinical episodes of malaria are important for the evolution of resistance because individuals who develop clinical symptoms are more likely to seek treatment; thus an increasing frequency of clinical episodes is associated with increasing drug pressure. Consequently, in this model clinically immune individuals represent a refuge for drug-sensitive pathogens because of lower treatment rates. The evolution of resistance is incorporated by assuming that individuals can be infected by either resistant or sensitive parasites.

### Population dynamics

It is assumed that both nonimmune and clinically immune individuals can be susceptible, infected with sensitive parasites, or infected with resistant parasites. The human population density in each state is denoted *S*_*i*_, *I*_*wi*_, *I*_*xi*_, where the subscripts *w *and *x *denote infections with drug-sensitive wild-type and resistant phenotypes, respectively, and the *i *subscript denotes the immune stage. The population size is normalized to one, and the population birthrate *B *is set equal to the per capita death rate of the human population, *μ*, so that the total population size stays constant.

### Entomology

Model notation follows Macdonald [32] and Smith and McKenzie [33]; *m *denotes the number of mosquitoes per human and *a *the human feeding rate (the number of bites on humans per mosquito per day). The instantaneous death rate is *g *(*e*^-*g *^is the probability of a mosquito surviving one day), and *n *is the number of days required for sporogony. Vectorial capacity (V), the number of infectious bites by a mosquito over its lifetime, is then given by the formula *V *= *ma*^2^*e*^-*gn*^/*g*.

The fraction *P *of bites on humans that infect a mosquito depends on the differing transmission intensities of the two-stages, *c*_1 _and *c*_2_, and the number of humans in each stage; thus *P *= *c*_1_(*I*_*w*1 _+ *I*_*x*1_) + *c*_2_(*I*_*w*2 _+ *I*_*x*2_). The sporozoite rate, or the fraction of infectious mosquitoes, is *aPe*^-*gn*^/(*g *+ *aP*). The entomological inoculation rate (EIR), the number of infectious bites per person per day, is calculated as the product of the human biting rate (*ma*) and the sporozoite rate (*aPe*^-*gn*^/(*g *+ *aP*)). The force of infection, or happenings rate (*h*), is *b*EIR, where *b*, the infectivity rate, measures the fraction of bites in humans that produce a patent infection. It follows that *h=*(*bVP*)/(*1 + sP*), where *s *= *a*/*g *is called the stability index, the number of bites on a human per vector per lifetime. The fraction of infections that are drug sensitive is *F*_*w *_*= *(*c*_1_*I*_*w*1 _+ *c*_2_*I*_*w*2_)/*P *and the fraction that are drug resistant is *F*_*x *_= (*c*_1_*I*_*x*1 _+ *c*_2_*I*_*x*2_)/*P*. Happenings rates for drug-sensitive and drug-resistant infections are *h*_*w *_*= F*_*w*_*h *and *h*_*x *_= *F*_*x*_*h*, respectively.

### Immunity acquisition and parasite clearance

Clinical immunity is assumed to develop in infected individuals after ten years. Once individuals gain immunity, protection is retained through biting and is lost at a faster rate (*γ*) than it is gained (*θ*). The values are based on a significant number of age-prevalence studies suggesting that children acquire immunity after approximately five to 10 years [29,30,34], and additional studies that suggest a strong role of biting in maintaining immunity, and a loss of immunity that occurs after exposure to infection is eliminated [35]. Because not all infections result in fever and other associated symptoms, it is assumed that clinical symptoms arise in infected individuals at a rate σ_*i *_and that a fraction *f*_*i *_are treated and cleared of parasites. Thus, existing infections are cleared by drugs at the rate *ρ*_*i *_= *f*_*i*_σ_*i*_. It is further assumed that a fraction of new infections *ξ*_*i *_in susceptible individuals develop clinical symptoms and are treated with drugs and cleared immediately prior to the development of gametocytes, thus precluding the possibility of transmission. In these individuals it is as if the infection never occurred.

Mutations conferring resistance to antimalarial drugs are likely to be disadvantageous to the parasite. For example, resistance to CQ in *P. falciparum *has been shown to have a fitness cost of approximately 25 percent *in vitro *[36] and 5 percent *in vivo *[37,38]. Although a fitness cost could conceivably occur at any or all stages of the parasite life-cycle, where it is implemented in this model is not particularly important for the transmission dynamics (though it could be important in other models), so it is assumed that resistant infections are cleared at a faster rate and therefore transmit for a relatively shorter period. Thus, infections clear naturally at rate *r*_*w *_when an individual is infected with a drug-sensitive phenotype and *r*_*x *_for drug-resistant infections.

### Equations

Based on the above assumptions, model dynamics are described by a simple set of coupled ordinary differential equations:

(1) $${\dot{S}}_{1}=B+\gamma S_{2}+I_{w1}(\rho_{1}+r_{w})+I_{x1}r_{x}-S_{1}(h_{w}(1-\xi_{1})+h_{x}+\mu )$$

(2) $${\dot{I}}_{w1}=S_{1}h_{w}(1-\xi_{1})-I_{w1}(\rho_{1}+r_{w}+\theta +\mu )$$

(3) $${\dot{I}}_{x1}=S_{1}h_{x}-I_{x1}(r_{x}+\theta +\mu )$$

(4) $${\dot{S}}_{2}=I_{w2}(\rho_{2}+r_{w})+I_{x2}r_{x}-S_{2}(h_{w}(1-\xi_{2})+h_{x}+\gamma +\mu )$$

(5) $${\dot{I}}_{w2}=S_{2}h_{w}(1-\xi_{2})+I_{w1}\theta -I_{w2}(\rho_{2}+r_{w}+\mu )$$

(6) $${\dot{I}}_{x2}=S_{2}h_{x}+I_{x1}\theta -I_{x2}(r_{x}+\mu )$$

**Figure 1:**
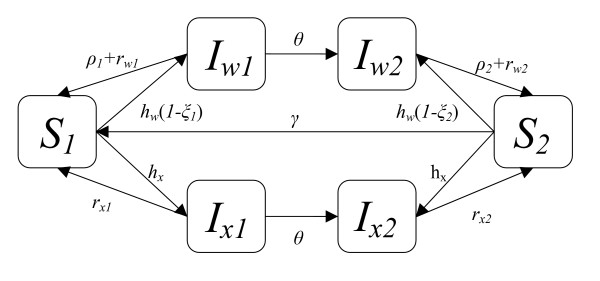
**SIS two-stage model**. Susceptible individuals (*S*) become infected (*I*) with wild-type infections at the rate *h*_*w*_(*1-ξ*_*i*_) and resistant infections at the rate *h*_*x*_, where *h*_*i *_is the happenings rate (see text) and *ξ*_*i *_is the rate at which new infections result in clinically manifested symptoms that are treated and resolved prior to the formation of gametocytes. Infected individuals naturally clear resistant infections at rate *r*_*xi*_, and they clear wild-type infections at the rate *ρ*_*i*_+*r*_*wi*_, where *ρ*_*i *_is the rate of drug treatment. Infected individuals acquire semi-immunity at rate *θ *and lose immunity at rate *γ *if they are no longer infected. Individuals die from all states at a background rate of μ and are born at rate *B *as nonimmune susceptibles (process not shown).

A diagram of the model is found in Figure 1.

## Results

The principal assumptions for the analysis were that immune individuals are less infectious to mosquitoes (*c*_2 _<*c*_1_) and less likely to develop clinical malaria and get treated (ρ_2 _< ρ_1 _and ξ_2 _< ξ_1_), and that parasites face a resistance-related fitness cost (*r*_*x *_> *r*_*w*_). While recent evidence has suggested that individuals with clinical-immunity may recover at faster rates [39], it was assumed that recovery from infection was independent of immune status in the base model. Based on these assumptions, the equilibrium of the system was determined without resistance and then the resulting spread of resistance was simulated across a range of transmission rates (from less than one to more than 600 infectious bites per person per year) using a set of baseline assumptions (Table 1).

**Table 1:** Baseline Parameter Values

Acquisition of clinical immunity	θ^-1^	10 years
Loss of clinical immunity	γ^-1^	2 years
Fraction of new infections that are treated and cleared	ξ_1_	0.3
	ξ_2_	0.01
Rate clinical symptoms arise (σ_*i*_) times Fraction treated (*f*_*i*_) equals rate existing infections are cleared by drugs (*ρ*_*i*_)	σ_1_*f*_1 _= ρ_1_	0.025(0.2) = 1/200
	σ_2_*f*_2 _= ρ_2_	0.01(0.2) = 1/500
Disease induced death rate	*m*	180/100000/year
Human feeding rate	*α*	0.3
Infectivity rate	*b*	0.8
Mosquito death rate	*g*	1/10
Number of days required for sporogony	*n*	10
Recovery rate	*r*_*w*_	1/(165/b)
	*r*_*x*_	*r*_*w*_(fitness cost)

**Figure 2:**
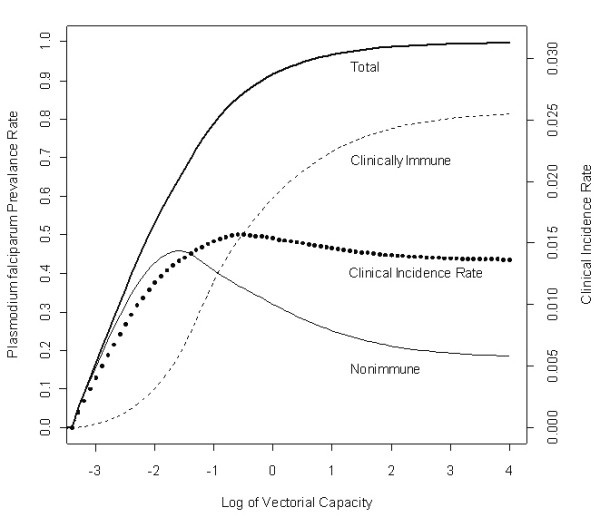
**Steady state infection level**. The proportion of people with *P. falciparum *infections increases extremely rapidly as vectorial capacity increases, but there is a large shift between those who are nonimmune and those who are immune. Semi-immune individuals mirror the trajectory of total infections as vectorial capacity increases and quickly become the large majority of infections. At low levels of vectorial capacity, the proportion of the population that is clinically immune remains extremely low. However, as vectorial capacity increases, the proportion of individuals that are clinically immune (nonimmune) increases (falls). The clinical incidence rate increases sharply at first but then plateaus as the proportion of the population that is clinically immune increases. The clinical incidence rate is defined at equilibrium as the sum of susceptible individuals who become infected with a wild-type phenotype and develop an immediate clinical infection plus individuals already infected who become symptomatic $\left(\sum_{i}\sum_{j}h_{w}S_{j}\xi_{j}+I_{i,j}s_{j}\right)$, where *i *and *j *are the phenotype of the infection and the immune stage, respectively.

At equilibrium without resistance, the results were consistent with other models of transmission. As vectorial capacity increases, the proportion of the population that is nonimmune, and thus subject to frequent attacks, increases sharply and then slowly decreases, while the proportion of immune individuals increases steadily until it plateaus at a fairly high level (Figure 2). As expected [24,31], the infected proportion of the population nears 100 percent at high transmission levels, though a significant portion of infected individuals are clinically immune. In addition, the results suggest that clinical episodes of malaria – a proxy for hospital admissions for malaria as well as severe malaria – plateau at intermediate levels of transmission, which is consistent with other research [30].

Because symptomatic individuals are more likely to use antimalarial drugs, the number of individuals experiencing a clinical episode affects the transmission dynamics by changing the drug pressure facing the parasite. Individuals that acquire clinical immunity, have reduced rates of clinical episodes [16-19], which reduces the rate of drug use. Though individuals will continue to receive antimalarial treatments throughout their lives, these treatments occur less frequently as immunity increases, and often can be unconnected to the peaks of parasitemia [3]. This reduction in the treatment rate reduces the resistance selection pressure, providing a refuge for drug-sensitive parasites. In previous epidemiological models of transmission, these immune individuals were not assumed to have a significant qualitative effect on the dynamics of the system, because young children were assumed to dominate transmission events [25]. However, clinically immune individuals are infectious and can significantly affect the dynamics of transmission when the distribution between sensitive and resistant infections is different in nonimmune and clinically immune individuals.

Distributional differences in the frequencies of infection types are driven by drug treatment rates, and these differences can be calculated as differences in the relative fitness of resistant parasites compared with sensitive parasites. Parasite fitness was calculated as the basic reproductive number (*R*_0_) of both the resistant and the sensitive parasites when the population is completely naïve (subscripted by 1) and fully immune (subscripted by 2). The *R*_0_-values of the sensitive parasites are

(7a) $$R_{0,w1}=\frac{bV(1-\xi_{1})}{\theta +\rho_{1}+r_{w}+\mu }\left[c_{1}+\frac{\theta c_{2}}{\rho_{2}+r_{w}+\mu }\right]$$

(7b) $$R_{0,w2}=\frac{bVc_{2}(1-\xi_{2})}{\rho_{2}+r_{w}+\mu }$$

and the R_0_-values of the resistant strains are

(8a) $$R_{0,x1}=\frac{bV}{\theta +r_{x}+\mu }\left[c_{1}+\frac{\theta c_{2}}{r_{x}+\mu }\right]$$

(8b) $$R_{0,x2}=\frac{bVc_{2}}{r_{x}+\mu }$$

Previous models suggested that the parasite with the highest *R*_0 _would be the most fit and should predominate [27,40]. That conclusion always holds when *r*_*x *_= *r*_*w*_, *c*_1 _= *c*_2_, and *ρ*_1 _= *ρ*_2_. However, differences in the drug treatment rates between clinically immune and nonimmune individuals can change the result, and it was found that resistant parasites may not spread even when they have the dominant *R*_0_. This can be explained by calculating the effective reproductive number of the resistant parasite, *R*_*x*_, where *R*_*x *_= *R*_0,*x*_*d*, and *d *is the fraction of the population that can be infected and will transmit the resistant infection. The effective reproductive number of the resistant parasite is

(9) *R*_*x *_= (*S*_1 _+ *S*_2_)((1 - *f*) *R*_0,*x*1 _+ *fR*_0,*x*2_)

where *f *is the fraction of the population that is clinically immune, and *S*_1 _and *S*_2 _are the proportion of the population that is susceptible and nonimmune, and susceptible and clinically immune, respectively.

**Figure 3:**
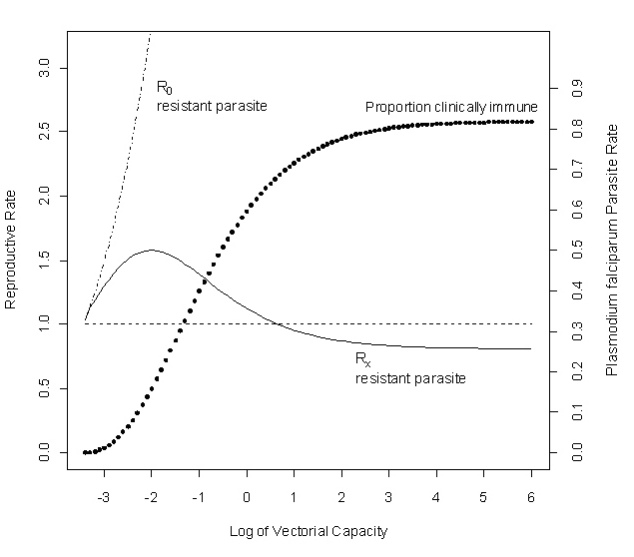
**Immunity reduces resistant parasite fitness**. The clinically immune class influences the ability of a resistant parasite to invade. Since individuals in this class do not progress to clinical malaria as often, they are treated less often; this creates a refuge for the wild-type parasites. As vectorial capacity increases, the clinically immune class is maintained at higher and higher levels until it becomes biologically impossible for the resistant parasite to spread. This paradigm exists because of the population of clinically immune individuals, without whom the resistant parasite would be able to spread at any vectorial capacity (as shown by the dashed line above).

The results suggest that as the transmission rate increases, the fraction of clinically immune individuals increases, significantly reducing the effective reproductive number of the resistant parasite. At certain parameter values it was even possible for the effective reproductive number of the resistant parasite to fall below 1 while the reproductive number of the sensitive parasite remained above 1, effectively abrogating the ability of resistant parasites to spread (Figure 3). Thus, at higher transmission levels it may be possible for the resistant parasite to have the dominant *R*_0 _but be unable to spread because of the population-wide level of immunity.

**Figure 4:**
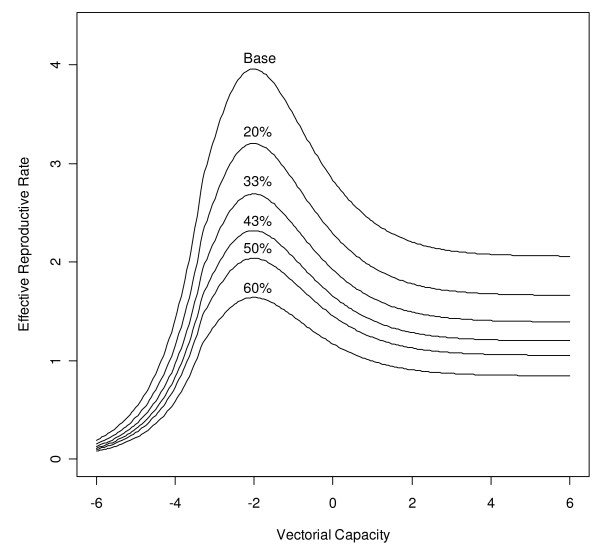
**Effective reproductive rate and fitness cost of resistance**. The higher the fitness cost of resistance (1 - *r*_*w*_/*r*_*x*_), the lower the absolute level of the effective reproductive number. Qualitatively, this suggests an important implication: it is always more difficult for the parasite to evolve (i.e., emerge and spread) resistance in higher-transmission settings.

Though the ability of the resistant parasite to spread is not always compromised, the generalizable result is that at all levels of fitness cost (including none), it is easier for resistant parasites to spread at lower levels of transmission (Figure 4). This reduction in the ability of individuals to transmit the resistant parasite suggests an important implication: it is relatively easier for resistance to spread in an area of lower transmission than in an area of higher transmission. This also helps explain, from an epidemiological perspective, why resistance in general tends to evolve faster in lower-transmission settings.

## Discussion

Simulations of the model found a significant relationship between malaria immunity and the ability of resistant parasites to spread from an initial locus. Previous epidemiological models of malaria transmission assumed that immune individuals were not infectious; however, the best available evidence in malaria suggests that immune individuals remain infectious, though they transmit less efficiently to mosquitoes [22-24,31] and the frequency and severity of clinical disease declines [16-19]. Because they remain infectious but asymptomatically infected, their relatively lower usage of antimalarials creates a natural refuge for sensitive parasites, similar to the way nontransgenic crops act as refugia for *Bt*-sensitive insects in agriculture [41]. Increases in the transmission rate result in concomitant increases in the proportion of clinically immune individuals, which increases the size of the refuge and reduces the ability of the resistant parasite to spread. This suggests an epidemiological rationale for the more likely emergence of resistance in low- or unstable-transmission settings.

The presence of a refuge of clinically immune individuals alters the ecological landscape that resistant parasites face, and introduces a mechanism for the coexistence of resistant and sensitive parasites. Simple models of directly transmitted infections [40], and a previous model of malaria transmission [27], suggested there was a critical threshold level of treatment above which resistance would fix and below which it would not spread. However, in those models, there was either no immunity, or immune individuals were assumed to be noninfectious and were, therefore, irrelevant for transmission and selection for resistance. Clinical immunity changes the criteria for determining the critical threshold so that the fraction of clinical episodes that are treated, as well as the level of immunity in the community, which determines the fraction of new infections that result in a clinical episode, are both important. Therefore, in populations where immunity has developed, the resistant parasite faces a different fitness landscape; they may not be the most fit, despite having the highest *R*_0_, which can prevent the parasite from either fixing or spreading. This result also suggests that differential treatment levels of host-groups can create differential ecological niches allowing the resistant and sensitive parasites to coexist [42].

### History of epidemiological models of malaria

There is a striking difference between the findings of this paper and a previously published epidemiological model of antimalarial resistance [27]. To explain the differences, a brief history of malaria models is warranted. A quantitative approach to malaria epidemiology organized around the parasite life-cycle was first described mathematically by Ross [43], and later revised by Macdonald to consider mosquito mortality during sporogony [32,44]. In the 1970s several major innovations in malaria modeling were first introduced and field-tested during a malaria control project in the Garki region of Nigeria [35]. The mathematical model of control developed for the Garki project considered the clearance of infection in hosts that have been infected by more than one parasite brood [20,45], and the development of blood-stage immunity and transmission-blocking immunity in the human host [20]. Despite a model that assumed parasite infections were complex and evidence that gametocytes remain present throughout life, the Garki model made a simplifying assumption that new infections remained infectious for a short period of time. Thereafter, infections persisted until the person either cleared the infection or developed immunity. The Garki model called this a semi-immune state. In this semi-immune state, humans could become re-infected, but they were never infectious. Mathematical epidemiologists later noted that individuals who were infected but not infectious were epidemiologically irrelevant for transmission, and the Garki model was simplified into an SIRS compartment model in which the dynamics of infection in the semi-immune population were replaced by a recovered and immune state with immune boosting [25,26]. The SIRS model was a familiar model to mathematical epidemiologists, and it has been the basis for many subsequent papers on malaria epidemiology, but the assumptions about immunity have been propagated and revised without much critical thought.

This paper is an attempt to demonstrate the importance of immunity in modeling the transmission dynamics of malaria with respect to the introduction of resistance. While the implications of the results are significant for drug policy, it is prudent to note that as with all models a number of simplifying assumptions have been made. In incorporating immunity, the process of immunity acquisition has been simplified so that individuals acquire immunity in a stepwise fashion after 10 years of continuous infection, and they lose immunity after two years of continuous non-infection. This is a simplification of the complicated process that is immunity. It ignores age effects, differences between immune individuals at older ages and younger ages, and faster acquisition of immunity, which may occur in areas of higher transmission [46] or after fewer challenges in low transmission areas [47]. While this is a simplification, evidence suggests that it gives a representation of reality that is close enough to make qualitative observations [29,30,34,35]. Despite the simplification of immunity, the basic model (without resistance) accords well with both field data and other models of transmission dynamics, and changes in these values did not change the qualitative results. Thus, the model, as formulated, likely captures the qualitative differences that exist between areas with different transmission levels, and is, hopefully, only the first step in more detailed modeling of the spread of antimalarial resistance which takes account of the importance of immunity.

## Conclusion

The link between the transmission rate and the probability that *de novo *mutants can both arise and spread is a critical issue for developing strategies to mitigate resistance emergence. Though it is impossible to be certain, historical analysis of the evolution of resistance has suggested that it originated in low or unstable transmission areas [2,3]. Quantitative population genetic models have supported this assertion, suggesting that there may be genetic reasons why resistance is less likely to evolve in high-transmission settings [3,9,11,12], but individual models may not always explain population-level phenomena. Epidemiological models, which can take account of specific population characteristics, have been hampered by the assumption that immune individuals play no qualitative role in the dynamics of transmission. Consequently, an epidemiological model could not offer an explanation for why resistance emerged outside Africa [27]. The model in this paper demonstrates that the existence of a refuge for drug-sensitive parasites in high-transmission areas can slow or prevent the evolution of resistance; this has enormous implications for the design of effective malaria control strategies.

Efforts to control malaria have been undermined by the emergence of resistance, which now exists to all known antimalarials except the artemisinin compounds. To help ensure a longer period of efficacy for artemisinin, the World Health Organization has proposed a global subsidy to comply with its mandate that all new artemisinin-based therapies be deployed as combinations [48]. One concern with this strategy is that increased use in high-transmission areas may engender resistance at a faster rate. The results as determined by this model, mitigates that concern, showing that the existence of a refuge of immune individuals makes this outcome highly unlikely. However, in areas with low transmission intensity, the relative paucity of immune individuals increases the risk that resistance will emerge. Because the emergence of resistance to an antimalarial in any country threatens the viability of the drug in all countries [49], areas of relatively lower transmission should be a focus in controlling the emergence of resistance. As a corollary, eliminating malaria from areas of low transmission intensity may have the global benefit of prolonging the effective lifetime of antimalarial drugs.

Although areas of low transmission should implement strategies to reduce the likelihood of resistance, high-transmission areas are also a concern because transmission intensity is reduced through mass distribution of insecticide-treated bed nets. The analysis in this paper suggests that among-host selection for resistance increases as functional immunity wanes and the refuge for drug-sensitive parasites shrinks or disappears. Thus, implementation of transmission reduction strategies should also include improved surveillance for drug resistance with resources to deploy appropriate containment strategies to prevent the geographical dissemination of resistance.

The intention of this study was to evaluate the qualitative importance of immunity in the transmission dynamics of malaria and its role in the development of resistance. Epidemiological models of malaria transmission, which can synthesize various factors about how emergence and spread are linked to the composition of the population, can be useful tools in determining optimal drug treatment strategies to extend the effective life of newly introduced therapies; however, they must take account of immune individuals and the refuge they provide for sensitive parasites.

## Authors' contributions

DLS and EYK developed the model. All authors contributed to the analysis and writing and have read and approved the final version of the manuscript.
